# Antibiotic strategies in the era of multidrug resistance

**DOI:** 10.1186/s13054-016-1320-7

**Published:** 2016-06-22

**Authors:** George Karam, Jean Chastre, Mark H. Wilcox, Jean-Louis Vincent

**Affiliations:** Infectious Disease Section, Louisiana State University School of Medicine, New Orleans, LA 70112 USA; Réanimation Médicale, Groupe Hospitalier Pitié-Salpêtrière, 75013 Paris, France; Department of Microbiology, Leeds Teaching Hospitals NHS Trust and University of Leeds, Leeds, LS1 3EX UK; Department of Intensive Care, Erasme Hospital, Université libre de Bruxelles, Route de Lennik 808, 1070 Brussels, Belgium

## Abstract

The rapid emergence and dissemination of antibiotic-resistant microorganisms in ICUs worldwide threaten adequate antibiotic coverage of infected patients in this environment. The causes of this problem are multifactorial, but the core issues are clear: the emergence of antibiotic resistance is highly correlated with selective pressure resulting from inappropriate use of these drugs. Because a significant increase in mortality is observed when antibiotic therapy is delayed in infected ICU patients, initial therapy should be broad enough to cover all likely pathogens. Receipt of unnecessary prolonged broad-spectrum antibiotics, however, should be avoided. Local microbiologic data are extremely important to predict the type of resistance that may be present for specific causative bacteria, as is prior antibiotic exposure, and antibiotic choices should thus be made at an individual patient level.

## Background

ICU patients are particularly at risk of developing infections with multidrug-resistant (MDR) organisms, which are more prevalent in this environment. Appropriate and adequate antibiotic coverage is essential in the treatment of these patients. In the present update, we will discuss the mechanisms of development of resistance, before highlighting some of the key issues related to antibiotic management and possible approaches to prevent further development of resistance.

## Epidemiology

**Fig. 1:**
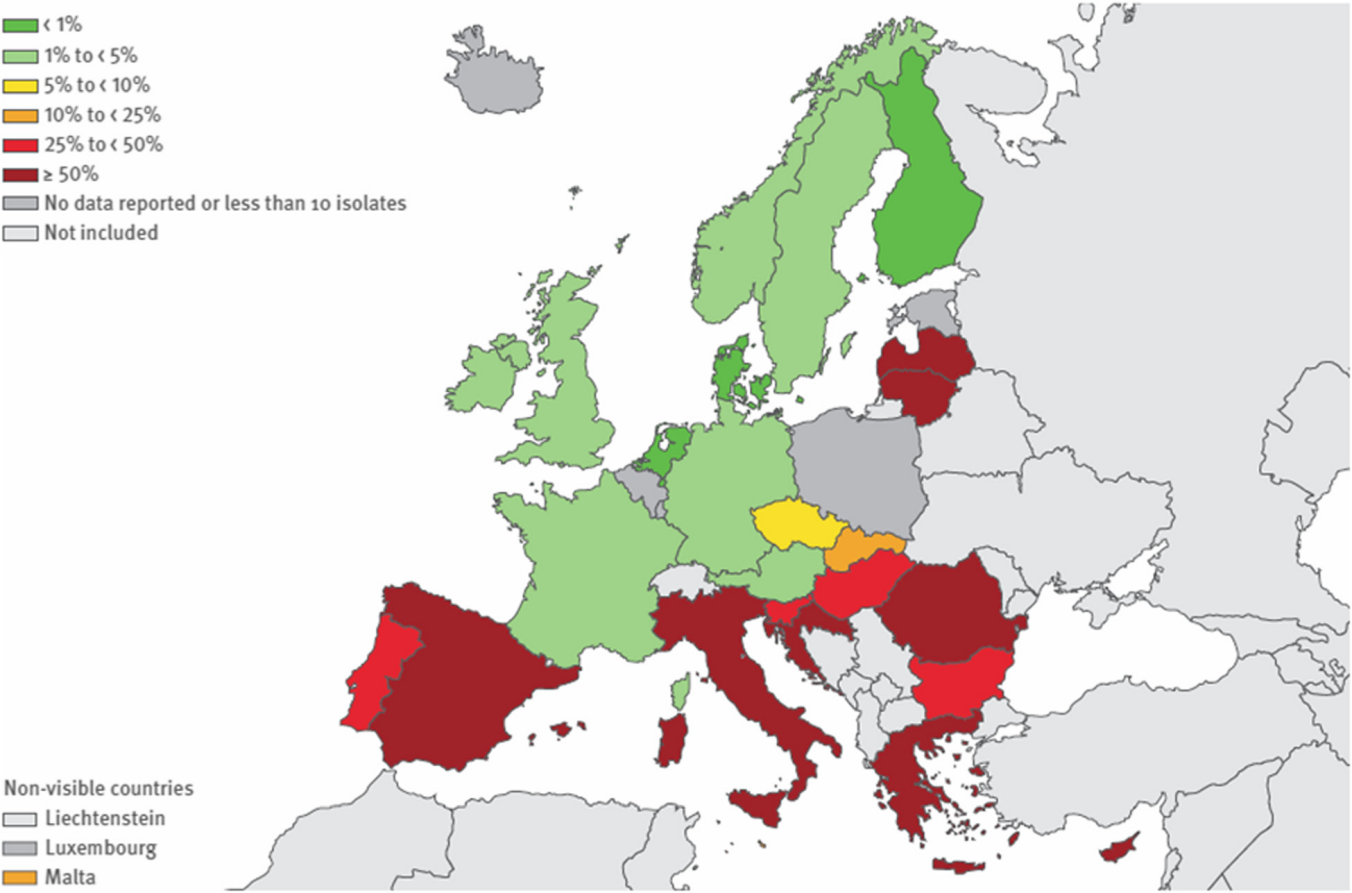
*Acinetobacter* species: percentage of invasive isolates with combined resistance to fluoroquinolones, aminoglycosides, and carbapenems. European Union/European Economic Area, 2014. From [1]

There is a general increase in the number of resistant microorganisms worldwide, although specific patterns vary considerably across countries. There has been a significant increase across Europe in the percentages of *Klebsiella pneumoniae* resistant to fluoroquinolones, third-generation cephalosporins, and aminoglycosides, as well as combined resistance to all three antibiotic groups [1]. *Escherichia coli* resistance to third-generation cephalosporins has also increased significantly, from 9.6 % to 12.0 % between 2011 and 2014 (population-weighted European Union/European Economic Area (EU/EEA) mean percentage of resistance) [1]. For *Acinetobacter* species, there is considerable variability in resistance rates, but high percentages (>50 %) of isolates with combined resistance to fluoroquinolones, aminoglycosides, and carbapenems have been reported from southern Europe (Fig. 1). Although the percentage of methicillin-resistant *Staphylococcus aureus* (MRSA) decreased between 2011 and 2014, this decrease was less pronounced compared with the previous 4-year period. In 2014, the EU/EEA population-weighted mean MRSA percentage remained high, with seven out of 29 reporting countries having MRSA percentages >25 %.

The increased prevalence of carbapenem-resistant *Enterobacteriaceae* (CRE), particularly in *K. pneumoniae* which has seen near untreatable infections occurring in an increasing number of hospitals, is also of concern. Greece, Italy, and Malta in Europe, the USA, South America, and Asia have notably been affected by these bacteria [2, 3]. Such is the level of threat that the US Centers for Disease Control and Prevention has named CRE as one of the top three most urgent antimicrobial-resistant challenges [4].

## Mechanisms of resistance

Resistance can occur in all types of pathogens encountered in the ICU setting, although Gram-negative bacteria are the most likely to exhibit resistance to multiple classes of antibiotics. The three most representative mechanisms of resistance to β-lactam antibiotics in Gram-negative bacteria are: destruction of antibiotics by β-lactamases; impermeability, including closure of porin channels in the bacterial cell wall (most notable as the mechanism of resistance to carbapenems for *Pseudomonas aeruginosa*); and extrusion of antibiotics by efflux pumps (which can lead to resistance to multiple classes of antibiotics). Analogous mechanisms of resistance occur with classes of antibiotics that are increasingly being used to manage infections due to bacteria resistant to β-lactam antibiotics (Table 1). By contrast to β-lactam antibiotics, which have their mechanisms of action and resistance located within the cell wall of the bacteria, the location of binding sites and modifying enzymes of the other antibiotic classes described in Table 1 are intracellular. Knowledge of this variability of action and resistance mechanisms can contribute to informed decisions in the selection of antimicrobial therapy for resistant organisms.

**Table 1:** Mechanisms of resistance in classes of antibiotics used to treat resistant pathogens

	Permeability [55]	Enzymatic destruction	Altered binding sites	Efflux [57]
β-lactams [32]	✓	✓	✓	✓
		β-lactamases	Penicillin-binding proteins	
Fluoroquinolones [56]	✓		✓	✓
			Alterations in DNA gyrase and topoisomerase IV	
			Protection by plasmid-mediated Qnr protein	
Aminoglycosides [58]	✓	✓	✓	✓
		Adenolyating and acetylating enzymes	30S ribosomal subunit	
Tetracyclines [59]	✓	✓	✓	✓
		Modification enzymes	70S ribosomal subunit	

Extended-spectrum β-lactamases (ESBLs) are broad-spectrum enzymes produced most characteristically by *E. coli*, *Klebsiella*, and *Proteus* species (Table 2). Representative of the ease with which resistance can occur, these enzymes may develop on the basis of a change in only one amino acid in the β-lactamases normally produced [5]. Despite the minimal structural change, ESBLs have the capacity to inactivate many broad-spectrum β-lactam drugs. It is noteworthy that use of third-generation cephalosporins and fluoroquinolones has been identified as a risk factor for selection of ESBLs [6, 7]. Of the clinically relevant attributes of these enzymes, ESBLs represent a classic example of a resistance mechanism in which in vitro susceptibility may not be consistently predictive of clinical efficacy.

**Table 2:** A clinical approach to β-lactamases

Type of β-lactamase	Classic microorganisms or types	Clinical prescribing challenges
Extended-spectrum β-lactamases	*Escherichia coli* *Klebsiella* species *Proteus* species	Variability of in vitro activity consistently predicting in vivo efficacy Collateral damage of selection by certain antibiotic classes
AmpC	*Serratia* species *Pseudomonas aeruginosa* Indole + *Proteus* *Citrobacter* species *Enterobacter* species	Collateral benefit of possibly preventing selection of resistance by limiting antibiotics with activity against certain bacteria (as an example, pseudomonal-sparing antibiotic regimens)
Carbapenemases	KPC (Ambler Class A) NDM (Ambler Class B) Oxa-type^a^ (Class D)	Selection risk by intensity and duration of prior antibiotic therapy Consideration of the role of combination therapy in treatment

^a^Most characteristically found in *Acinetobacter*

*KPC* *Klebsiella pneumoniae* carbapenemase, *NDM* New Delhi metallo-β-lactamase (found in *Enterobacteriaceae*)

By contrast to the plasmid-mediated production of ESBLs, AmpC β-lactamases most classically are chromosomally-mediated and occur in such important ICU pathogens as *P. aeruginosa* and *Enterobacter* species. In recent years, however, plasmid-mediated AmpC β-lactamases have been identified in pathogens such as *E. coli* and *K. pneumoniae*. Influencing the risk that antibiotics pose for the selection of infection caused by resistant pathogens is the fact that one in every 10^6^–10^7^ organisms with the characteristic potential for AmpC β-lactamase production (listed in Table 2) has a spontaneous mutation that allows it to produce this enzyme [8]. These mutant strains are not naturally competitive and do not, therefore, overgrow to destroy the sensitive nonmutant flora. However, when antibiotics are given that destroy the sensitive flora, the resistant mutant strains can proliferate and establish themselves as the predominant pathogens. In such a scenario, injudicious use of broad-spectrum agents may lead to the development of clinical resistance during therapy.

There has been considerable recent publicity about a study from China that has described the first strains of *E. coli* with plasmid-mediated colistin resistance [9]. Although there are concerns about the use of colistin, including toxicity and dosing uncertainties, it is one of a very few alternatives that can be used in some cases to treat infections caused by CRE. This novel mechanism of colistin resistance, previously only chromosomally mediated, is of grave concern given that strains harboring the plasmids are already widely prevalent in animals in China, and also (albeit lesser so) in some clinical isolates.

Carbapenemase production by Gram-negative bacteria is one of the most concerning patterns of resistance encountered in the ICU because it is associated increasingly with resistance to all presently marketed antibiotics. Using the Ambler classification of β-lactamases—in which there are Classes A, B, C, and D—carbapenemases occur within three of the four classes. Class B β-lactamases have a metallo base, and the initial carbapenemases described clinically were Class B metallo-enzymes. A representative example from clinical practice of a Class B metallo-enzyme is the New Delhi metallo-β-lactamase, which is found in certain *Enterobacteriaceae*. Ambler Class A β-lactamases are serine based and include the majority of ESBLs. Carbapenemases produced by *K. pneumoniae* were identified within this Ambler class and subsequently characterized as *K. pneumoniae* carbapenemases (KPCs). Gaining increasing importance today are the Class D serine-based carbapenemases, with the most classic being the oxa-type enzymes produced by organisms such as *Acinetobacter* species. Even though the name carbapenemase identifies the ability of these enzymes to inactivate carbapenem antibiotics, carbapenemases are not specific for carbapenem antibiotics but have the ability to hydrolyze β-lactams of all classes [10].

## Links between antibiotic prescribing and resistance

Most of the time, antibiotics do not create resistance per se. Antibiotic resistance determinants have been circulating within the microbial genome for millennia, largely predating the manufacture and use of antibiotics by human beings [11, 12]. This was conclusively demonstrated by D’Costa et al. [12] using targeted metagenomic analyses of rigorously authenticated ancient DNA from 30,000-year-old permafrost sediments and the identification of a highly diverse collection of genes encoding resistance to β-lactam, tetracycline, and glycopeptide antibiotics, very similar to the genes currently expressed by bacteria in healthcare-associated infections. The emergence and dissemination of resistant microorganisms during antimicrobial therapy is thus essentially driven by the selection of the small fraction of naturally resistant bacteria that exist in all microbiota, because of the pressure they exert on the susceptible microorganisms, giving a survival advantage to the cells that are intrinsically resistant to the antimicrobial agent(s) used. In ICU patients, this phenomenon is not only operational at the level of the infected site but also at the level of the digestive tract microbiota and other commensal floras, where the huge number of bacteria present may very rapidly promote the emergence of drug-resistant microorganisms. Even if these bacteria do not themselves cause disease, they can easily share these resistance genes with bacteria that do, through direct exchange of DNA (by conjugation or extrachromosomal plasmid DNA). One recent study confirmed that short exposure to imipenem in ICU patients was followed by a significant increase in carriage of imipenem-resistant Gram-negative bacilli [13]. The risk of acquisition was 5.9 times higher in patients who received only 1–3 days of imipenem treatment compared with controls, and increased to 7.8 times higher in those who received longer treatments.

Numerous studies in human and veterinary medicine have shown a correlation between consumption of antimicrobials and resistance in bacteria isolated from infected humans [14–19]. Recent studies have also confirmed the direct effect of antibiotic use in selecting resistant organisms at the individual level. For example, Malhotra-Kumar et al. [20] demonstrated in a double-blind, randomized trial performed in a large group of healthy volunteers that macrolide exposure for 7 days led directly to the emergence of resistance in the oral streptococcal flora.

In addition to the selection pressure they exert on susceptible bacteria, antibiotics can influence antibiotic resistance through several other mechanisms, including changes in cell permeability and efflux or alterations in the antibiotic target, and horizontal transfer of resistance genes [21]. Many antibiotics, even at very low concentrations that cannot kill susceptible bacteria, induce the formation of reactive oxygen species (ROS), which can damage bacterial DNA, increasing genetic variability [22]. Selection of hypermutable clones is another undesirable consequence that can enhance resistance development.

The potential to select resistant strains of *P. aeruginosa* with antibiotics that have activity against *P. aeruginosa* has been demonstrated repeatedly in the medical literature [23, 24]. What has evolved from this risk of selecting resistance is the concept of stratifying therapy for infections based on the likelihood of the presence or absence of *P. aeruginosa* as the etiologic agent of the infection, and this has been a core consideration in multiple clinical guidelines [25, 26].

There are numerous reports of carbapenemase outbreaks linked to various classes of antibiotics. In contrast to establishing a link to only one specific class of antibiotics, there is an evolving body of medical literature suggesting an important relationship between prior antimicrobial therapy and the subsequent identification of carbapenemase-producing bacteria. In a 4-year case–control study of 102 patients, the only covariate independently associated with CRE in all multivariate analyses was the cumulative number of prior antibiotic exposures [27]. A 26-month case–control study (96 ESBL-carbapenem-resistant *K. pneumoniae* and 55 ESBL-carbapenem-sensitive *K. pneumoniae*) from Greece identified both prior cumulative exposure to antibiotics and increasing duration of prior treatment as risk factors [28]. Antibiotic treatments shown in this study to be associated with the isolation of carbapenemases were therapy with β-lactam/β-lactamase inhibitor or with a combination of fluoroquinolone and carbapenem. These data are consistent with previous reports that no particular class of antibiotic is the predominant predisposing factor for selection of carbapenemase production, but that it is rather the intensity and duration of antibiotic therapy which are the most important variables in creating the milieu in which carbapenemase-producing bacteria are selected.

## Management: patient stratification and antibiotic choices

Perhaps the greatest challenge imposed by MDR Gram-negative bacteria is attainment of the appropriate degree of balance between efficacy and ecology. It is well recognized that there are significant increases in mortality rates when antibiotic therapy is delayed [29, 30], challenging the clinician to prescribe therapy broad enough to cover all of the most likely pathogens. What is simultaneously recognized is the collateral damage of selecting resistant bacteria that can occur with prolonged receipt of broad-spectrum antibiotics [31].

Three important categories can influence antimicrobial choices: patient characteristics; risk factors for infection with specific pathogens; and severity of illness. Of the demographic data of patients with infections, advanced age and comorbid illnesses have been associated with decreased reserve and, hence, an increase in mortality. Contact with the health care system is an important patient characteristic that influences the broadness of initial antibiotic therapy. Previous hospitalization (within the past 30 days as a defining feature of hospital-acquired infection and within the past 90 days as a defining feature of healthcare-associated infection) increases the risk of infection by a resistant pathogen that was acquired in the healthcare environment. Invasive procedures, even those occurring in the outpatient setting, increase the risk of colonization by MDR organisms, with such colonization serving as an antecedent predisposition to infection with MDR microorganisms. Local microbiologic data are extremely important in predicting the type of resistance that may be present in the etiologic agent causing a clinical infection. It is important to recognize that such local data may vary from unit to unit within a hospital, and when possible unit-specific data are optimal. In recognition of the fact that resistance can rapidly develop within a practice environment, it is important that microbiologic data should be as current as possible. Antibiograms reporting data that are from a previous year may be many months behind what is presently occurring. Very important is the role of prior antibiotic exposure, recognizing the risk of eliminating normal flora and allowing the selection of resistant bacteria. Although various timelines have been suggested in the medical literature about what defines “recent” antibiotic therapy, within the previous 90 days is a consistently cited number. A second element of importance in stratification regarding antibiotic therapy is the likely type of microorganism. As noted previously, risk stratification related to *P. aeruginosa* has been included in multiple clinical guidelines [25, 26]. Of all the variables that influence stratification of patients into a category requiring coverage of MDR bacteria, severity of illness may be the most important. As severity of illness increases, the margin for error in initial antimicrobial therapy diminishes and this requires consideration of coverage against multiple patterns of bacterial resistance.

Since the reviews by Jacoby and Munoz-Price [32] and by Paterson and Bonomo [5] listing carbapenems as drugs of choice for infections caused by ESBL-producing bacteria, this class of antibiotics has been considered first-line therapy for such infections. This recommendation is influenced by the fact that ESBL-producing pathogens are often resistant to fluoroquinolones and aminoglycosides since resistance mechanisms for these classes of antibiotics are often carried on the same large plasmids that contain the genetic elements for ESBL production. Paradoxically, even though carbapenems are β-lactam agents, they are stable in the presence of ESBLs. More recently and in response to a large volume of infections in Spain caused by ESBL-producing bacteria, a group of investigators from that country conducted a post hoc analysis of patients with bloodstream infections due to ESBL-producing *E. coli* from six published prospective cohorts [33]. In this trial, mortality and length of hospital stay were compared in patients treated with either amoxicillin–clavulanic acid (AMC) or piperacillin–tazobactam (PTZ) versus those receiving a carbapenem. In this analysis, the infection source was the urinary or biliary tract in two-thirds of the patients. The results suggest that AMC and PTZ are suitable alternatives to carbapenems for treating patients with bloodstream infections due to ESBL-producing *E. coli* if there is in vitro activity. However, the variable in vitro susceptibility of ESBL-producing bacteria to β-lactam/β-lactamase inhibitor combinations warrants consideration before these agents are used as empiric therapy for serious infections.

Further addressing the question of whether PTZ is clinically as efficacious as carbapenems in the treatment of bacteremia caused by ESBL-producing organisms, investigators compared 14-day mortality when PTZ versus a carbapenem was used as empiric therapy (defined in this trial as antibiotic therapy administered to a patient before their ESBL status was known) in a cohort of 331 patients with ESBL bacteremia who received definitive therapy with a carbapenem [34]. The adjusted risk of death was 1.9 times higher for patients receiving empiric PTZ compared with empiric carbapenem (95 % confidence interval 1.07–3.45).

It is important to acknowledge that the data regarding the role of PTZ in the treatment of ESBL-producing bacteria may not be an accurate predictor of how new β-lactamase inhibitors (e.g., avibactam or relebactam) or new combinations (e.g., ceftolozane/tazobactam) may perform in the treatment of these infections. Ceftolozane/tazobactam in combination with metronidazole was shown to be noninferior to meropenem in a multinational, double-blind randomized phase 3 trial of adult patients hospitalized with complicated intraabdominal infections [35]. Of the isolated pathogens, 7.2 % were ESBL-producing *Enterobacteriaceae* and for this group of patients clinical cure rates were 95.8 % in the ceftolozane/tazobactam group and 88.5 % in the meropenem group. Similar findings were reported from a randomized trial of adult patients hospitalized with complicated lower urinary tract infections or pyelonephritis, in which 7.6 % of the isolated pathogens were ESBL-producing *Enterobacteriaceae* [36].

In a recent review, Harris et al. [37] suggested three clinical scenarios in which β-lactam/β-lactamase inhibitors might be appropriate instead of using a carbapenem in the treatment of infections caused by ESBL-producing organisms: urinary tract infections (including with bacteremia); nonurinary tract infections in which the isolate is susceptible at a low minimum inhibitory concentration (MIC); and when adequate source control has been achieved.

In a retrospective study of monomicrobial bacteremia caused by ESBL-producing organisms, patients were definitively treated with cefepime if there was in vitro activity to that antibiotic or with a carbapenem [38]. Patients who received cefepime as definitive therapy were more likely to have clinical failure, and the survival analysis consistently found that individuals who received cefepime therapy had a lower survival rate. Based on these observations, the authors concluded that cefepime empirical therapy was inferior to carbapenem in the treatment of patients with bacteremia due to cefepime-susceptible ESBL-producing bacteria. It is noteworthy that the cefepime breakpoint for susceptibility in this study was ≤8 μg/ml, which may have overstated the susceptibility rate, compared with the more recent breakpoints of ≤2 μg/ml from the Clinical and Laboratory Standards Institute (CLSI) and ≤1 μg/ml from the European Committee on Antimicrobial Susceptibility Testing (EUCAST), which would result in lower rates of susceptibility [39].

A major challenge in the ICU is achieving adequate therapy for infections caused by carbapenemase-producing bacteria. An important early observation regarding therapy of such infections was that monotherapy with an agent like polymyxin might not provide optimal efficacy. In an analysis of 15 studies and case reports of the therapy of infections caused by KPCs, the success of polymyxin monotherapy was 14 % in contrast to 73 % with polymyxin combinations [40]. Considerations regarding such failure include the lack of optimal dosing of polymyxin (potentially due to variables such as augmented renal clearance in patients with severe illness) versus the lack of an additive or even synergistic effect that might occur with combination therapy. Early data suggested a potential role for carbapenems as a therapeutic option if the MIC of the infecting organism was ≤4 mg/l and if the carbapenem was given in combination with another active agent [41]. In an observational study from Greece regarding the treatment of infections due to carbapenemase-producing *K. pneumoniae*, patients who received a carbapenem in combination with other active agents had a mortality rate of 19.3 % if the carbapenem MIC of the infecting organism was ≤8 μg/ml versus a mortality rate of 35.5 % if the MIC was >8 μg/ml [42]. An overall observation in this trial was that mortality was higher in patients who received monotherapy than in those treated with combination regimens. In a retrospective analysis of the data from this trial, the lowest mortality was observed in patients treated with a carbapenem-containing regimen. However, a recent analysis of 12 retrospective cohort studies or case series, two prospective observational studies, and two randomized controlled trials showed no difference in mortality between colistin alone and colistin/carbapenem combination therapy [43]. Several clinical trials are ongoing to further assess this issue (ClinicalTrials.gov NCT01732250 and NCT01597973). Until these data are available, a major challenge for the clinician will be to interpret existing data—most of which are observational or retrospective—and make clinical decisions regarding the therapy for such infections. Important considerations in those analyses will be the microbiologic data of the organism (including the specific carbapenem MICs), the timing of the administered antibiotics, the effectiveness of therapy based on pharmacokinetic/pharmacodynamic considerations, and the potential for additive or even synergistic effects with combination therapy.

## Prevention

A fundamentally important challenge in clinical medicine is how to control or prevent antibiotic resistance. Several strategies may be beneficial.

### Rapid diagnostics

Rapid treatment of sepsis is associated with decreased mortality in patients with serious infections, but diagnosis of sepsis can be difficult and delayed in critically ill patients. Hence broad-spectrum empirical antibiotics are often started before microbiology results are available, resulting in some patients receiving unnecessary antibiotic treatment. More rapid, culture-independent identification methods are being developed [44], which, in addition to providing quicker identification of the pathogen causing an infection, can also rapidly identify the susceptibility patterns of the organism. The benefits of such information are twofold: if the organism possesses resistance mechanisms, appropriate therapy based on susceptibilities can be more quickly accomplished; and, based on susceptibility information, the duration of broad-spectrum therapy may be limited. By enabling earlier, accurate diagnosis of infection and of the causative microorganism(s), these tests may help optimize antibiotic prescriptions, and in so doing potentially reduce selection pressure and thus resistance.

### Colonization prevention

Embracing the principle that colonization is the antecedent event leading to clinical infection, the clinician is poised to incorporate an important infection prevention opportunity into clinical practice. In an observational cohort study in two ICUs with endemic carbapenemase-producing *Enterobacteriaceae*, patients were screened with perineal swabs at admission and twice-weekly thereafter [45]. Patients colonized with carbapenemase-producing *Enterobacteriaceae* had a 1.8 times greater hazard of dying in the ICU than noncolonized patients, primarily because of an increased length of stay. In a study designed to prevent colonization and infection by KPC-producing *Enterobacteriaceae* in four long-term acute-care hospitals with high endemic KPC prevalence, a bundled intervention was tested using a stepped-wedge design [46]. Patients were screened for rectal KPC colonization on admission and every other week. Contact isolation and geographic separation of KPC-positive patients was implemented in ward cohorts or single rooms. All patients were bathed daily with chlorhexidine gluconate. Healthcare workers were educated, and their adherence was monitored. During the intervention period of the study, the incidence rate of KPC colonization fell from 4 to 2 acquisitions per 100 patient-weeks (*p* = 0.004 for linear decline). Compared with the preintervention period, there were decreases in the isolation of KPC in any clinical culture, KPC bacteremia, and all-cause bacteremia. The demonstration that prevention of colonization can have significant clinical benefits in the era of carbapenemase infections has significant implications for ICU and non-ICU settings.

### Heterogeneity of antibiotic usage

The basis for this approach was noted in the mid-1990s through a program of informatics at LDS Hospital in Salt Lake City, UT, USA [47]. Subsequently, other groups found that patterns of antibiotic use in which the same antibiotic was given repeatedly (i.e., homogeneity) were associated with higher rates of resistance than when there was variability in the antibiotics given by the same prescriber among patients (i.e., heterogeneity) [48]. Several studies have evaluated the role of empiric antibiotic rotation protocols in reducing development of antibiotic resistance. In a surgical ICU, Bennett et al. [49] reported that monthly cycling of four antibiotics (PTZ, imipenem/cilastin, ceftazidime, and ciprofloxacin) as the primary antibiotic to treat suspected Gram-negative infections was associated with an overall improvement in the antibiotic susceptibility profile of Gram-negative organisms compared with the medical ICU in the same hospital where cycling was not performed. The 2007 antimicrobial stewardship guideline from the Infectious Diseases Society of America/Society of Healthcare Epidemiology of America [50] stated that there were “insufficient data to recommend the routine use of antimicrobial cycling as a means of preventing or reducing antimicrobial resistance over a prolonged period of time”.

Ironically, formal cycling may impose antibiotic selection pressures during those periods when a particular agent is preferred. However, increased diversity of prescribing has been shown to correlate with reduced levels of resistance [51]. The challenge therefore remains how increased antibiotic heterogeneity can be achieved in a way that delivers benefits rather than risks.

### Short duration antibiotic courses

Since the classic paper in the 1980s elucidating the concept that clinical resistance in Gram-negative pathogens occurs on the basis of selection by antibiotics of spontaneously mutant strains of bacteria which possess resistance mechanisms [8], there has been increasing awareness about the importance of appropriately limiting the duration of antibiotic therapy. This strategy has taken two important forms in recent years. One has been de-escalation of therapy, in which the spectrum of empiric antibiotics is narrowed when microbiological data become available to minimize the selective pressure of antibiotics. A second strategy has been antimicrobial stewardship, which targets the duration of therapy as an important means of achieving optimal clinical outcomes. Current guidelines recommend a course of 7–10 days for most severe infections [52], although some recent data support the use of shorter courses in certain infections, such as intraabdominal infections [53]. Many clinicians, however, remain hesitant about prescribing fewer fixed days of antibiotics for patients with severe bacterial infection, and prefer to customize antibiotic duration based on the clinical course of the disease and/or using serial determinations of a biological marker of infection, such as procalcitonin (PCT). Adapting the antimicrobial treatment duration to PCT kinetics has been demonstrated as useful in several randomized trials targeting patients with acute respiratory infection [54]. Nevertheless, PCT kinetics should only be used as a tool to support clinical judgment.

## Conclusion

There is no doubt that we are now faced with greater antibiotic resistance challenges than ever before, limiting treatment options for patients with severe infections. The rate of development of new antimicrobial agents has failed to keep pace with the “ingenuity” of bacteria to mutate and become resistant to antibiotics. We have to adapt to this threat by reducing unnecessary antibiotic prescribing, both qualitatively and quantitatively. We need to optimize control measures to minimize the risk of spread of resistant bacteria, and we have to find novel ways to detect pathogens early. These approaches will help prevent the spread of MDR pathogens and could enable us to direct last-line (and in some cases, narrow-spectrum) antibiotics more effectively to those patients who need them most, rather than the current “broad-spectrum is best” approaches.

## Abbreviations

AMC: amoxicillin–clavulanic acid
CRE: carbapenem-resistant *Enterobacteriaceae*
ESBL: extended-spectrum β-lactamase
EU/EEA: European Union/European Economic Area
KPC: *Klebsiella pneumoniae* carbapenemase
MDR: multidrug-resistant
MIC: minimum inhibitory concentration
MRSA: methicillin-resistant *Staphylococcus aureus*
PCT: procalcitonin
PTZ: piperacillin–tazobactam
ROS: reactive oxygen species
